# Research on the quality markers of antioxidant activity of Kai-Xin-San based on the spectrum–effect relationship

**DOI:** 10.3389/fphar.2023.1270836

**Published:** 2023-12-27

**Authors:** Xiaoxiao Shan, Xuan Yang, Dawei Li, Lele Zhou, Shaogang Qin, Junying Li, Wenkang Tao, Can Peng, Jinming Wei, Xiaoqin Chu, Haixuan Wang, Caiyun Zhang

**Affiliations:** ^1^ School of Pharmacy, Institute of Pharmacokinetics, Anhui University of Chinese Medicine, Hefei, Anhui, China; ^2^ Center for Xin’an Medicine and Modernization of Traditional Chinese Medicine of IHM, Grand Health Research Institute of Hefei Comprehensive National Science Center, Anhui University of Chinese Medicine, Hefei, China; ^3^ Anhui Education Department (AUCM), Engineering Technology Research Center of Modernized Pharmaceutics, Hefei, Anhui, China; ^4^ Anhui Province Key Laboratory of Pharmaceutical Preparation Technology and Application, Anhui University of Chinese Medicine, Hefei, Anhui, China; ^5^ Anhui Genuine Chinese Medicinal Materials Quality Improvement Collaborative Innovation Center, Hefei, Anhui, China; ^6^ Anhui Academy of Chinese Medicine, Anhui University of Chinese Medicine, Hefei, China; ^7^ Hefei Food and Drug Inspection Center, Hefei, Anhui, China

**Keywords:** Kai-Xin-San, Q-marker, HPLC fingerprint, spectrum–effect relationship, antioxidant activity

## Abstract

**Background:** Kai-Xin-San (KXS) is one of the classic famous traditional Chinese medicine prescriptions for amnesia, which has been applied for thousands of years. Modern pharmacological research has found that KXS has significant therapeutic efficacy on nervous system diseases, which is related to its antioxidant activity. However, the antioxidant material basis and quality markers (Q-makers) of KXS have not been studied. Objective: The objective of this study is to explore the Q-makers of antioxidant activity of KXS based on spectrum-effect relationship.

**Methods:** Specifically, the metabolites in KXS extracts were identified by UPLC-Q-Exactive Orbitrap MS/MS. The fingerprint profile of KXS extracts were established by high-performance liquid chromatography (HPLC) and seven common peaks were identified. Meanwhile, 2, 2-diphenyl-1-picrylhydrazyl (DPPH) test was used to evaluate the free radical scavenging ability of KXS. The spectrum-effect relationship between its HPLC fingerprint and DPPH free radical scavenging activity was preliminarily examined by the Pearson correlation analysis, grey relation analysis (GRA), and orthogonal partial least squares discrimination analysis (OPLS-DA). Further, the antioxidant effect of KXS and its Q-makers were validated through human neuroblastoma (SH-SY5Y) cells experiment.

**Results:** The results showed that 103 metabolites were identified from KXS, and the similarity values between HPLC fingerprint of twelve batches of KXS were greater than 0.900. At the same time, the results of Pearson correlation analysis showed that the peaks 8, 1, 14, 17, 18, 24, 16, 21, 15, 13, 6, 5, and 3 from KXS were positively correlated with the scavenging activity values of DPPH. Combined with the results of GRA and OPLS-DA, peaks 1, 3, 5 (Sibiricose A6), 6, 13 (Ginsenoside Rg1), 15, and 24 in the fingerprints were screen out as the potential Q-makers of KXS for antioxidant effect. Besides, the results of CCK-8 assay showed that KXS and its Q-makers remarkably reduced the oxidative damage of SH-SY5Y cells caused by H2O2. However, the antioxidant activity of KXS was decreased significantly after Q-makers were knocked out.

**Conclusion:** In conclusion, the metabolites in KXS were successfully identified by UPLC-Q-Exactive Orbitrap MS/MS, and the Q-makers of KXS for antioxidant effect was analyzed based on the spectrum-effect relationship. These results are beneficial to clarify the antioxidant material basis of KXS and provide the quality control standards for new KXS products development.

## Graphical Abstract

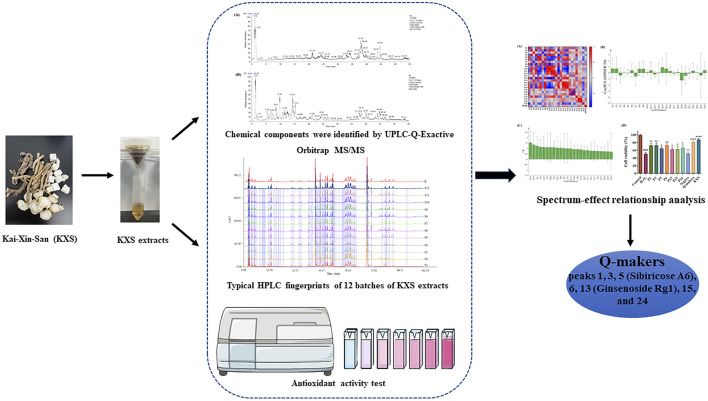

## 1 Introduction

In recent years, more and more traditional Chinese medicine (TCM) prescriptions have been unearthed, some of which are known as classic famous prescriptions because of their wide application, precise efficacy, and obvious characteristics (Li et al., 2015). Kai-Xin-San (KXS) is one of the classic famous prescriptions for amnesia, recorded by Sun Simiao in Bei Ji Qian Jin Yao Fang during the Tang dynasty (652 AD) (Wang et al., 2019a; Fu et al., 2020). KXS is composed of Polygalae Radix (*Polygala tenuifolia* Willd.), Ginseng Radix et Rhizoma (*Panax ginseng* C. A. Mey.), Acori Tatarinowii Rhizoma (*Acorus tatarinowii* Schott), and Poria cocos (*Poria cocos* (Schw.) Wolf) (Qiong et al., 2016). As a tranquilizing medicine, Polygalae Radix has the effect of calming the mind, improving intelligence, and regulating the heart and kidney (Jiang et al., 2021). It was widely applied in treating Alzheimer’s disease (AD), depression, epilepsy, and other central nervous disorders (Zhao et al., 2020). Meanwhile, Ginseng Radix et Rhizoma is a great tonic medicine for benefiting Qi and invigorating vitality, with pharmacological effects which include improving learning and memory, stimulating the central nervous system, anti-tumor effects, and strengthening the immune system (Shi et al., 2013; Jiang et al., 2019; Yu et al., 2020). Additionally, studies have reported that Acori Tatarinowii Rhizoma has various pharmacological effects, including antioxidant and anti-depression properties, protecting nerve cells, alleviation of learning and memory impairment, and anti-myocardial ischemia (Zhang et al., 2015; Wen et al., 2023). In addition to regulating gut microbiota, *Poria cocos* has anti-inflammatory and antioxidant pharmacological activities in treating diseases (Fang et al., 2021; Xu et al., 2022). The compatible combination of these four drugs makes KXS have the advantages and characteristics in treating neurological diseases (Cao et al., 2018).

So far, many pharmacological studies have shown that KXS significantly improves depression and AD through anti-oxidation and anti-inflammation, and by inhibiting apoptosis (Guo et al., 2019; Hu et al., 2020a; Jiao et al., 2022). The metabolites of KXS are the real material basis for its efficacy (Yi et al., 2020). Among the metabolites in KXS, many of them have antioxidant activity (Xiao et al., 2020; Zhong et al., 2020; Lyu et al., 2021; Balakrishnan et al., 2022; He et al., 2022; Tang et al., 2022). For example, 3,6′-disinapoylsucrose, a metabolite of Polygalae Radix, has a protective effect against Aβ_1-42_-induced pathological damages, which may be associated with the reduction of Aβ deposition and anti-oxidation (Tang et al., 2022). Likewise, Ginsenoside Rg1, β-asarone, and pachymic acid possess significant antioxidant effects (Zhong et al., 2020; Balakrishnan et al., 2022; He et al., 2022). As is known, oxidative stress is one of the pathogenic mechanisms of AD (Zhao et al., 2020), as well as an important factor in the pathogenesis of depression and anxiety (Xiao et al., 2020; Zhong et al., 2020; Lyu et al., 2021; Balakrishnan et al., 2022; He et al., 2022; Tang et al., 2022). It is necessary to study the antioxidant activity of KXS and its material basis with a key marker. However, the overall spectrum–effect relationship between the antioxidant efficacy and the metabolites of KXS is still unclear.

The quality control of TCM is always a challenge due to the complexity of the metabolites and the ambiguity of the pharmacodynamic material basis. Fortunately, the quality marker (Q-marker) of TCM has fundamentally improved the idea and model of the quality assessment from the transmission and traceability, specificity, effectiveness, testability, and compound formula compatibility of metabolites (Ren et al., 2020). Proposed by Academician Liu, the Q-marker refers to the metabolites inherent in TCM or formed in the process of processing and preparation, which reflects the safety and effectiveness of TCM (Zhang et al., 2021a). As the intrinsic metabolites in botanical drugs and Chinese medicine products, Q-markers are crucial for establishing TCM quality assessment standards and traceability. In recent years, there have been several technical means to investigate the Q-markers of TCM (Xie et al., 2019; Zhang et al., 2021a). Combining the advantages of ultra-performance liquid chromatography (UPLC) and high-resolution mass spectrometry (MS), UPLC-MS/MS can produce secondary mass spectral information (including precursor and fragment ions) for aiding structural inference with good selectivity, sensitivity, mass accuracy, and exclusive detection (Yang et al., 2021). Among them, UPLC-Q-Exactive Orbitrap MS/MS conducts qualitative and quantitative analyses of complex botanical or biological sample metabolites using high selectivity of the quadrupole to parent ions and high resolution of precise mass numbers of the orbital ion trap (Orbitrap) with a low matrix effect (Wang et al., 2019b).

In recent years, the fingerprint profile established by high-performance liquid chromatography (HPLC) has been comprehensively, qualitatively, and quantitatively developed for the identification and quality evaluation of the complex multi-component TCM system (Fan et al., 2020). HPLC fingerprints can effectively separate the diverse metabolites in TCM and screen its characteristic metabolites based on the spectrum–effect relationship (Chen et al., 2020; Fan et al., 2020). As a stable free radical, 2,2-diphenyl-1-picrylhydrazyl (DPPH) is commonly used to measure the free radical scavenging activity of antioxidants and find the high antioxidant metabolites in TCM (Yu et al., 2021).

After the identification of the metabolites in KXS, the spectrum–effect relationship can be further determined based on HPLC fingerprinting and the DPPH scavenging activity of KXS, which is helpful to screen Q-markers for the antioxidant activity of KXS. Therefore, the metabolites in KXS were first identified by UPLC-Q-Exactive Orbitrap MS/MS in this study. Then, the HPLC fingerprint of KXS was established under gradient elution conditions with 12 batches of raw material, and their free radical scavenging activity was assessed by the DPPH test. The spectrum–antioxidant effect relationship of KXS was analyzed by the Pearson correlation analysis, gray relational analysis (GRA), and orthogonal partial least squares discriminant analysis (OPLS-DA). Finally, the Q-markers for the antioxidant effect of KXS were screened by the spectrum–effect relationship analysis and *in vitro* validated by the CCK-8 method. This study provided a solid research foundation for screening KXS antioxidant metabolites and establishing the quality evaluation standards for the future development of KXS products.

## 2 Materials and methods

### 2.1 Materials

Polygalae Radix, Ginseng Radix et Rhizoma, Poria cocos, and Acori Tatarinowii Rhizoma were obtained from Anhui Jingdao Co., Ltd. (Anhui, China), which were cultivated in their original authentic producing origins (Supplementary Table S1). These species were identified by Professor Can Peng in the Anhui University of Chinese Medicine, according to the 2020 edition of the Chinese Pharmacopoeia.

The reference substances of Sibiricose A5 (batch number: PS000944; purity: 99.41%) and Sibiricose A6 (batch number: PS000945; purity: 96.62%) were acquired from Chengdu Push Bio-Technology Co., Ltd. (Chengdu, China). β-Asarone (batch number: MUST-21082510; purity: 99.76%) and α-asarone (batch number: MUST-21092810; purity: 99.94%) were bought from Chengdu Man Site Co., Ltd. (Chengdu, China). Polygalaxanthone III (batch number: 111850-202006; purity: 95.30%), 3′,6-disinapoylsucrose (batch number: 111848-202006; purity: 96.50%), and Ginsenoside Rg1 (batch number: 110703-202034; purity: 94.00%) were obtained from National Institutes for Food and Drug Control (Beijing, China). DPPH was purchased from Shanghai Yuanye Co., Ltd. (Shanghai, China), and the H_2_O_2_ solution was obtained from Sigma (Shanghai, China). Mass spectrometric acetonitrile was purchased from Shanghai Macklin Biochemical Technology Co., Ltd. (Shanghai, China). HPLC-grade methanol and acetonitrile were obtained from Thermo Fisher Scientific (Massachusetts, United States).

### 2.2 Qualitative analysis of metabolites in KXS

#### 2.2.1 Preparation of the sample solution

In this study, 12 combinations were selected by the random number table method from 81 permutations of Polygalae Radix, Ginseng Radix et Rhizoma, Poria cocos, and Acori Tatarinowii Rhizoma with different producing origins (Zhou et al., 2022). Then, Polygalae Radix, Ginseng Radix et Rhizoma, Poria cocos, and Acori Tatarinowii Rhizoma were separately crushed, sieved, and mixed with a weight ratio of 1: 1: 2: 1 to obtain KXS (Zhang and Zeng, 2020). The KXS extract was obtained by adding 2 g of KXS into 25 mL of 75% methanol and extracting by ultrasound for 30 min (Shang et al., 2023). The extract was filtered through a 0.22-μm-pore-size membrane before testing.

#### 2.2.2 UPLC-Q-Exactive Orbitrap MS/MS analysis

The chromatographic and mass spectrometry conditions were performed in the Orbitrap Exploris 120 high-resolution mass spectrometer (Thermo Scientific, Bremen, Germany) using an ACQUITY UPLC BEH C18 column (1.7 μm, 2.1 × 100 mm, Waters). The mobile phase consisted of acetonitrile (A) and 0.1% formic acid solution (B) at a flow rate of 0.2 mL/min. The gradient protocol was as follows (A: B, v/v): 0–9 min, 7%–14% A; 9–13 min, 14%–16% A; 13–19 min, 16%–19% A; 19–23 min, 19%–23% A; 23–32 min, 23%–36% A; 32–37 min, 36%–38% A; and 37–45 min, 38%–46% A. The column temperature was 30°C, and the volume of sample injection was 2 μL.

The ESI ion source temperature was 120°C, and the scanning range of MS was m/z 50–1,500 Da. The capillary voltage was 3.5 kV in the positive ion collection mode and −2.5 kV in the negative ion collection mode. The cone voltage was 50 V, the ion source temperature was 110°C, the cone gas flow was 50 L/h, the atomization gas (N2) flow was 600 L/h, and the solvent removal temperature was 350°C. Leucine enkephalin was used as the calibration solution with an accurate mass number.

### 2.3 HPLC fingerprint method

#### 2.3.1 Chromatographic conditions

HPLC analysis was performed using the Thermo Fisher Ultimate 3000 high-performance liquid chromatograph with a UV detector and chromatographic column of Thermo-Acclaim^TM^120-C18 (250*4.6 mm, 5 μm). The mobile phase consisted of acetonitrile (A) and 0.1% aqueous phosphoric acid solution (B). The HPLC elution conditions were optimized as follows (A: B, v/v): 0–10 min, 95%–85% B; 10–20 min, 85%–84% B; 20–31 min, 84%–82% B; 31–33 min, 82%–77% B; 33–52 min, 77%–60% B; 52–70 min, 60%–48% B; 70–76 min, 48%–27% B; 76–93 min, 27%–20% B; 93–100 min, 27%–5% B; and 100–102 min, 5% B. The detection wavelength of the UV detector was set at 230 nm, and the column temperature was maintained at 30°C. The volume of sample injection was 10 μL, and the flow rate was 1.0 mL·min^−1^.

#### 2.3.2 Preparation of the standard solution

The seven reference standards (Sibiricose A5, Sibiricose A6, Polygalaxanthone III, 3′,6-disinapoylsucrose, Ginsenoside Rg1, β-asarone, and α-asarone) were precisely weighed and dissolved in 75% methanol. Subsequently, the final concentrations of Sibiricose A5, Sibiricose A6, Polygalaxanthone III, 3′,6-disinapoylsucrose, Ginsenoside Rg1, β-asarone, and α-asarone were 0.50, 0.50, 0.12, 0.20, 0.87, 0.09, and 0.10 mg·mL^−1^, respectively, obtained by diluting the aforementioned solution.

#### 2.3.3 Validation of the methodology

For the specificity of the method, the blank solvent (75% methanol) was injected into the HPLC system for determination, according to the chromatographic conditions given in “2.3.1”. Method precision was evaluated by six successive injections of the KXS sample solution (Sample S1). Similarly, six sample solutions were prepared according to “2.3.2”, and the repeatability was estimated by the method in “2.3.1”. The stability of the KXS sample solution was analyzed within 1 day (0, 2, 4, 8, 12, and 24 h) (Hu et al., 2020b).

#### 2.3.4 Similarity evaluation of HPLC fingerprints and peak identification

The obtained fingerprints (S1–S12) were imported into the Similarity Evaluation System for Chromatographic Fingerprint of TCM (Version 2004A, Beijing, China) and analyzed. With the aid of the similarity evaluation system for the TCM chromatographic fingerprint, the HPLC fingerprints of KXS were matched automatically (Goodarzi et al., 2013). The reference fingerprint was generated using the median method, and the similarity values between the reference fingerprint and the chromatogram of 12 batches of KXS were calculated.

Seven standard solutions and the mixed standard solution were injected into the HPLC system, respectively. According to the retention time (RT) of seven reference substances, the chromatographic peaks of the seven metabolites in the KXS extracts were identified.

### 2.4 DPPH free radical scavenging activity test

The experiment was performed according to the procedure described by Xiao et al. (2022). Briefly, 2 mL of the DPPH free radical solution (0.04 g/L in 75% methanol) and 2 mL of the blank solvent were mixed in the test tube, and allowed to react in the dark for 30 min. Finally, the absorbance value of the sample was measured using a UV spectrophotometer (λ = 517 nm) and recorded as A1. Similarly, different concentrations of the sample solution (6.25–200 μg/mL) were added into the DPPH free radical solution, respectively. After placing in the dark for 30 min, the absorbance of the samples was measured at λ = 517 nm as A2. At the same time, different concentrations of the sample solution and blank solvent were also mixed, and absorbance was measured as A3. The following equation was applied to compute the free radical scavenging rate (K).

$$K\;\left(\%\right)=\left[1‐\left(A2‐A3\right)/A1\right]*100$$

In this experiment, the median scavenging concentration (SC_50_), that is, the drug concentration with a DPPH free radical scavenging rate of 50%, was calculated by non-linear regression analysis.

### 2.5 Spectrum–effect relationship analysis

The spectrum–effect relationship between the HPLC fingerprints and the DPPH free radical scavenging activity of KXS extracts was examined by the Pearson correlation analysis, GRA, and OPLS-DA. In the Pearson correlation model, the areas of 25 common peaks in the HPLC fingerprints were set as one variable, and the SC_50_ values of KXS scavenging DPPH free radicals were used as another variable to analyze the correlation coefficients. Meanwhile, the DPPH free radical scavenging effect of KXS was used as the reference sequence for GRA, and the common characteristic peaks of the fingerprint profile (X1–X25) were listed as the comparative sequence. The contribution of each common peak to the efficacy was determined by comparing the gray correlation between the comparative sequence and the reference sequence. Similarly, OPLS-DA was also analyzed by SPSSAU (SPSS Inc., United States) and SIMCA (Umetrics Inc., Sweden).

### 2.6 Preparation of the sample and Q-marker-knockout samples of KXS

Under the HPLC conditions for separating KXS, the Q-marker sample and the sequential knockout samples of KXS were collected separately, according to the spectrum–effect relationship and their RT (Liu et al., 2021). The eluents were rotationally evaporated to remove the mobile phase, and the Q-marker sample, the target knockout metabolites, and the negative samples were obtained.

### 2.7 *In vitro* cell experiment

Here, human neuroblastoma (SH-SY5Y) cells were provided by the China Center for Type Culture Collection (Shanghai, China) and grown in complete DMEM at 37°C with 5% CO_2_. Then, the SH-SY5Y cells were seeded in 96-well plates at a concentration of 10^4^ cells/well and treated with 200 μmol/L H_2_O_2_ for 24 h to cause oxidative damage (Gai et al., 2019). To validate the antioxidant activity of KXS and its Q-markers, the Q-markers and sequential knockout samples of KXS were evenly dispersed in complete DMEM with 200 μg/mL (concentration of KXS) and added to the H_2_O_2_-induced SH-SY5Y cells for 24 h incubation. Following the treatment period, a solution containing WST-1 was added and incubated for another 1 h. The spectrophotometric absorbance was measured at 450 nm, and the cell viability was calculated using the following equation:

$$\text{Viability of cells }\left(\%\right)=\left(A_{\text{sample}}‐A_{\text{blank}}\right)/\left(A_{\text{control}}‐A_{\text{blank}}\right)\times 100\;\%$$

### 2.8 Statistical analysis

The metabolite identification was analyzed by Xcalibur 2.1 and Compound Discoverer 3.0 software applications. The dose–response curves were analyzed with the probit model by SPSS statistics software (SPSS 23.0, SPSS Inc., United States) (Ma et al., 2018). SPSSAU (SPSS Inc., United States) and SIMCA (Umetrics Inc., Swedish) were used for the Pearson correlation analysis, GRA, and OPLS-DA analysis (Liu et al., 2019). The data from the cell experiment were analyzed using GraphPad Prism 9.0 software. Student’s *t*-test and one-way analysis of variance (ANOVA) were applied for statistical analysis.

## 3 Results

### 3.1 Phytochemical analysis of KXS

**FIGURE 1:**
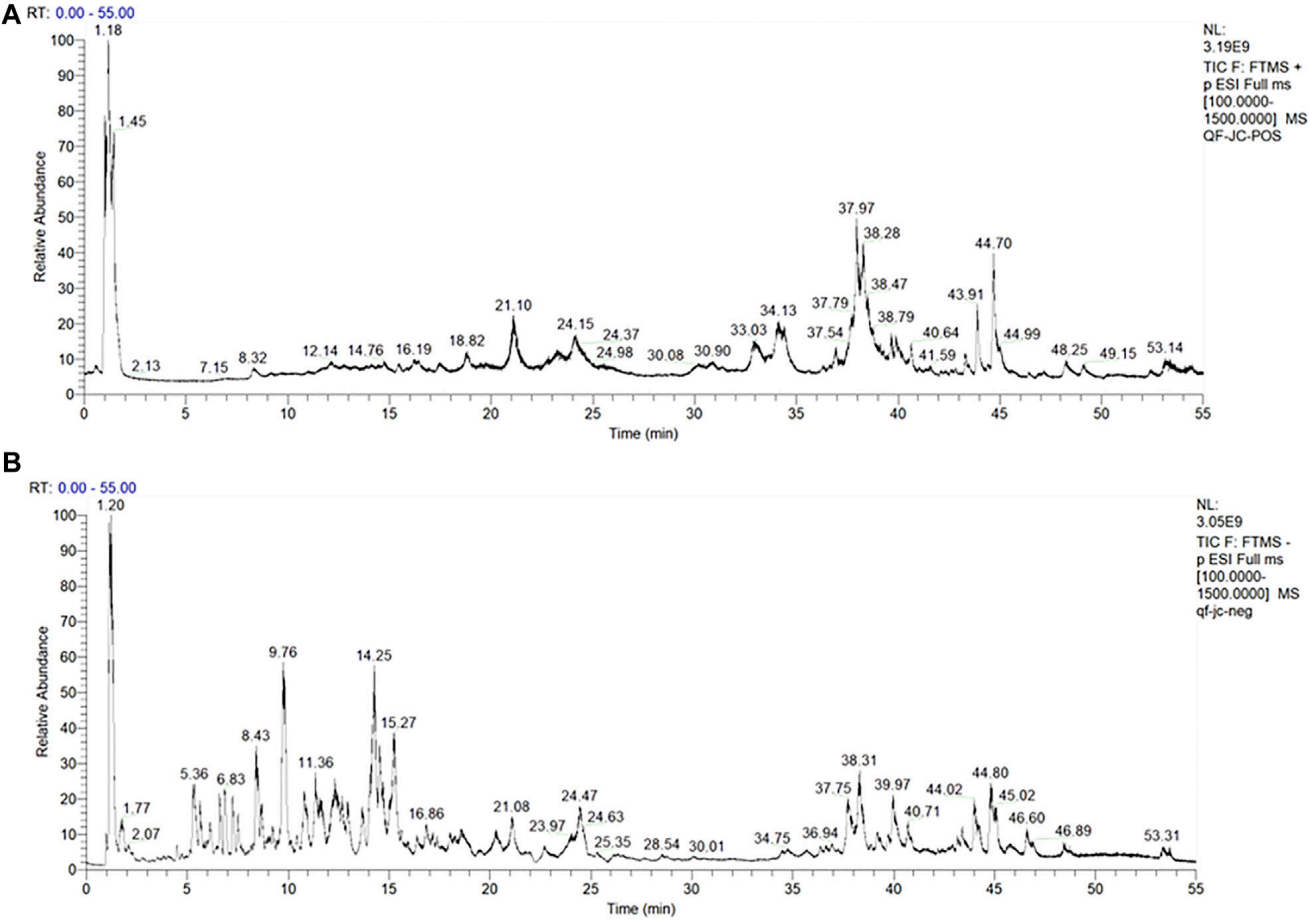
TIC of KXS extracts acquired by UPLC-Q-Exactive Orbitrap MS/MS in the positive **(A)** and negative **(B)** ion modes.

The total ion chromatograms (TICs) of the KXS extract acquired by UPLC-Q-Exactive Orbitrap MS/MS in the positive and negative ion modes are shown in Figure 1, and the identified metabolites are listed in Supplementary Table S2. In total, we identified 103 metabolites in KXS by comparison with the database and related literature studies (Wang et al., 2019b; He et al., 2022). Among them, Sibiricose A5, Sibiricose A6, Polygalaxanthone III, and 3′,6-disinapoylsucrose from Polygalae Radix, Ginsenoside Rg1 from Ginseng Radix et Rhizoma, and β-asarone and α-asarone from Acori Tatarinowii Rhizoma were reported to have antioxidant activity (Kumar et al., 2012; Shi et al., 2015; Liu et al., 2018a; Balakrishnan et al., 2022). Here, these successfully identified antioxidant metabolites can be further used for spectrum–effect relationship analysis.

### 3.2 HPLC method validation

The results of specificity showed that the blank solvent did not interfere with the determination, namely, the method had good specificity. The relative standard deviation (RSD) of RT and the average peak area (APA) of common peaks were calculated. The RSD of method precision, reproducibility, and storage stability of sample solutions within 24 h appeared less than 3.00%. All test results demonstrated that this chromatographic method was reliable in the KXS fingerprint analysis (Zhang et al., 2021b).

**FIGURE 2:**
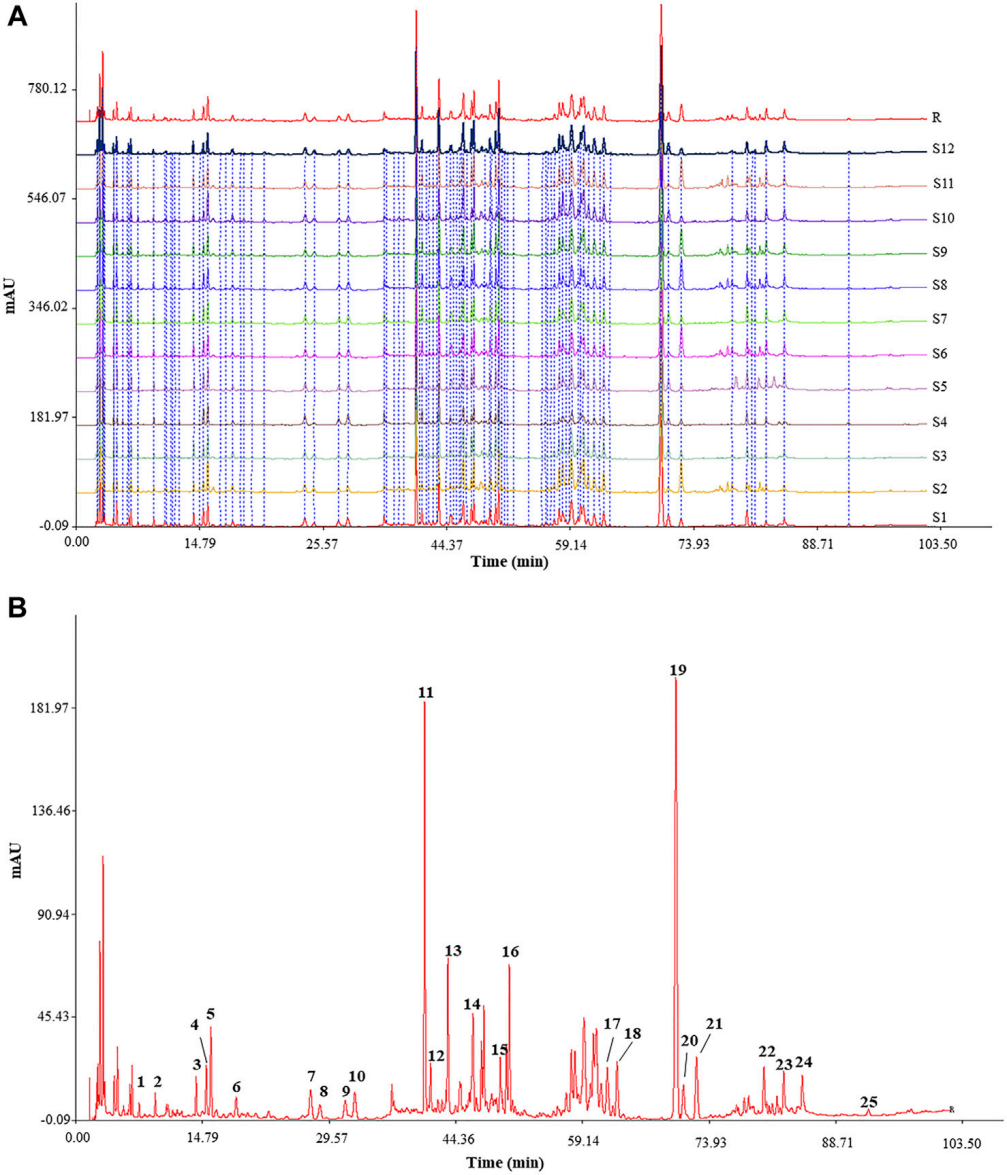
Typical HPLC fingerprints of 12 batches of KXS extracts **(A)** and reference chromatogram generated from KXS extracts. **(B)** Peak 4: Sibiricose A5; Peak 5: Sibiricose A6; Peak 10: Polygalaxanthone III; Peak 11: 3′,6-disinapoylsucrose; Peak 13: Ginsenoside Rg1; Peak 19: β-asarone; Peak 21: α-asarone.

The characteristic HPLC fingerprints of 12 batches of KXS extracts are shown in Figure 2A, while the developed reference atlas was displayed in Figure 2B. As common peaks, 25 peaks with good stability were chosen. Based on the RT of seven reference standards, the common peaks of 4, 5, 10, 11, 13, 19, and 21 were identified as Sibiricose A5, Sibiricose A6, Polygalaxanthone III, 3′,6-disinapoylsucrose, Ginsenoside Rg1, β-asarone, and α-asarone, respectively. APA of the 25 common peaks of 12 batches of KXS extracts and the RSD value are shown in Table 1. These results revealed the contents of 25 metabolites represented by common peaks varied greatly in the S1–S12 KXS extracts.

**TABLE 1:** Average peak area of common peaks of KXS extracts.

Peak	t_R_/min	Metabolite	The average peak area of every common peak from 12 patches of sample												
*No*.			S1	S2	S3	S4	S5	S6	S7	S8	S9	S10	S11	S12	RSD (%)
1	7.397	Unknown	0.602	1.091	0.707	0.463	0.513	0.660	0.567	1.020	1.047	1.067	1.031	0.421	34.46
2	9.277	Unknown	1.662	1.081	1.107	1.443	1.615	1.750	1.457	1.657	0.893	1.128	1.140	1.100	21.83
3	14.063	Unknown	2.885	1.742	3.537	0.450	2.605	3.111	2.522	2.404	2.831	1.678	2.834	2.786	33.36
4	15.217	Sibiricose A5	4.476	2.451	3.176	4.226	4.645	4.714	4.432	4.711	3.091	3.147	3.217	3.110	21.63
5	15.753	Sibiricose A6	6.296	9.898	6.665	5.543	6.458	6.649	6.157	6.510	6.433	9.892	6.659	6.327	20.20
6	18.733	Unknown	2.086	3.096	2.862	1.613	2.180	2.505	1.932	2.402	2.376	2.991	2.548	2.497	17.89
7	27.363	Unknown	4.539	5.448	3.763	5.442	4.716	4.925	4.612	4.880	4.013	4.625	4.205	4.157	11.38
8	28.477	Unknown	1.597	2.719	2.688	1.493	2.013	2.190	2.001	2.173	2.790	2.659	2.890	2.700	20.66
9	31.397	Unknown	2.523	1.835	2.121	4.417	3.706	3.800	2.536	3.801	2.832	1.813	2.971	2.115	30.40
10	32.517	Polygalaxanthone III	4.278	2.985	2.864	5.764	4.686	4.691	4.363	4.682	3.049	3.012	3.257	2.979	25.00
11	40.687	3′,6-Disinapoylsucrose	28.171	23.158	26.030	29.630	29.426	29.762	28.306	29.397	25.955	21.789	27.366	25.942	9.60
12	41.377	Unknown	4.221	3.306	3.729	4.086	5.010	4.955	4.186	4.910	4.290	2.237	4.533	3.637	19.38
13	43.403	Ginsenoside Rg1	11.556	12.121	12.286	11.313	12.063	12.534	11.791	12.407	12.743	9.524	13.366	12.282	7.90
14	46.330	Unknown	8.280	12.589	10.963	6.187	8.918	9.076	8.510	9.008	11.056	14.114	11.491	11.076	21.44
15	49.503	Unknown	5.146	4.120	4.493	3.954	4.828	5.112	5.204	5.012	4.896	3.871	5.223	4.498	10.57
16	50.567	Unknown	9.408	12.037	11.847	7.066	12.138	11.692	9.539	11.503	12.041	11.647	12.522	12.000	14.50
17	61.987	Unknown	5.695	8.785	8.113	3.967	6.935	7.100	6.434	7.016	8.618	7.177	8.938	7.972	19.73
18	63.130	Unknown	5.792	8.012	7.282	3.782	5.773	5.984	6.374	5.893	7.059	7.833	7.331	7.257	18.06
19	69.993	β-Asarone	55.069	62.117	58.490	49.801	56.915	64.624	54.039	65.998	59.729	53.886	58.990	53.864	8.33
20	70.873	Unknown	6.011	2.893	6.943	3.288	6.538	3.151	6.041	3.306	2.975	6.331	2.838	5.915	36.46
21	72.400	α-Asarone	3.603	15.710	3.611	2.767	3.924	15.820	3.419	15.882	13.861	3.100	14.748	3.222	73.68
22	80.287	Unknown	5.695	3.137	3.997	4.066	8.332	6.019	5.608	6.221	4.288	3.728	5.117	4.166	28.52
23	82.563	Unknown	1.919	3.413	1.955	3.440	6.371	6.591	6.780	6.731	7.863	6.762	6.783	6.829	343.14
24	84.737	Unknown	3.169	2.762	3.053	1.303	9.569	6.333	7.295	6.632	7.522	7.945	6.101	7.251	44.25
25	92.453	Unknown	0.786	0.713	0.774	1.227	0.692	0.751	1.116	0.741	0.696	0.717	0.780	1.117	22.78

### 3.3 Similarity analysis of HPLC fingerprints

To evaluate the similarity of HPLC fingerprints, 12 batches of KXS sample solutions were analyzed by comparing each sample fingerprint with the reference fingerprint (R) (Wang et al., 2021). As shown in Table 2, the similarity values between each HPLC fingerprint of KXS were in the range of 0.911–0.999. These similarity values were greater than 0.900, meaning that the quality of KXS was relatively consistent and stable.

**TABLE 2:** Calculation results of the fingerprint similarity of S1–S12 KXS.

	S1	S2	S3	S4	S5	S6	S7	S8	S9	S10	S11	S12	R
R	0.990	0.976	0.991	0.964	0.980	0.991	0.991	0.990	0.992	0.976	0.992	0.992	1.000
S12	0.985	0.961	0.993	0.952	0.972	0.971	0.991	0.969	0.981	0.980	0.981	1.000	
S11	0.970	0.977	0.976	0.936	0.967	0.990	0.973	0.988	0.998	0.963	1.000		
S10	0.958	0.975	0.976	0.916	0.949	0.949	0.966	0.947	0.965	1.000			
S9	0.969	0.977	0.976	0.937	0.966	0.991	0.975	0.990	1.000				
S8	0.975	0.964	0.968	0.955	0.969	0.999	0.974	1.000					
S7	0.994	0.950	0.986	0.968	0.977	0.975	1.000						
S6	0.975	0.965	0.969	0.954	0.970	1.000							
S5	0.973	0.936	0.965	0.952	1.000								
S4	0.977	0.911	0.958	1.000									
S3	0.990	0.967	1.000										
S2	0.950	1.000											
S1	1.000												

### 3.4 Results of DPPH free radical scavenging activity

The dose–response curves and their SC_50_ values of the DPPH free radical scavenging activity are listed in Table 3. It can be seen that these SC_50_ values of 12 batches of KXS extracts fluctuated within the range of 3.69–37.32 μg·mL^−1^. Meanwhile, the concentration–response curves demonstrated that the DPPH free radical inhibitory capacity of the KXS extracts (at a concentration of 6.25–200 μg/mL) possessed a good concentration–effect relationship (Bettencourt et al., 2019). Although KXS extracts can effectively scavenge DPPH free radicals, their antioxidant activities need further pharmacological relevant study.

**TABLE 3:** Concentration–effect curves and SC_50_ values of 12 batches of KXS extracts in scavenging DPPH free radicals.

Sample	Concentration–response curves	SC_50_ (μg·mL^−1^)
1	Probit (P) = −0.594–0.450 (lg concentration)	7.44
2	Probit (P) = −0.535–0.409 (lg concentration)	3.69
3	Probit (P) = −0.603–0.441 (lg concentration)	7.16
4	Probit (P) = −0.701–0.436 (lg concentration)	10.92
5	Probit (P) = −0.656–0.381 (lg concentration)	6.65
6	Probit (P) = −0.623–0.410 (lg concentration)	6.68
7	Probit (P) = −0.653–0.442 (lg concentration)	9.80
8	Probit (P) = −0.783–0.413 (lg concentration)	12.60
9	Probit (P) = −0.824–0.415 (lg concentration)	14.88
10	Probit (P) = −0.767–0.530 (lg concentration)	21.37
11	Probit (P) = −0.879–0.584 (lg concentration)	37.32
12	Probit (P) = −0.681–0.499 (lg concentration)	13.86

### 3.5 Results of Pearson correlation analysis

**FIGURE 3:**
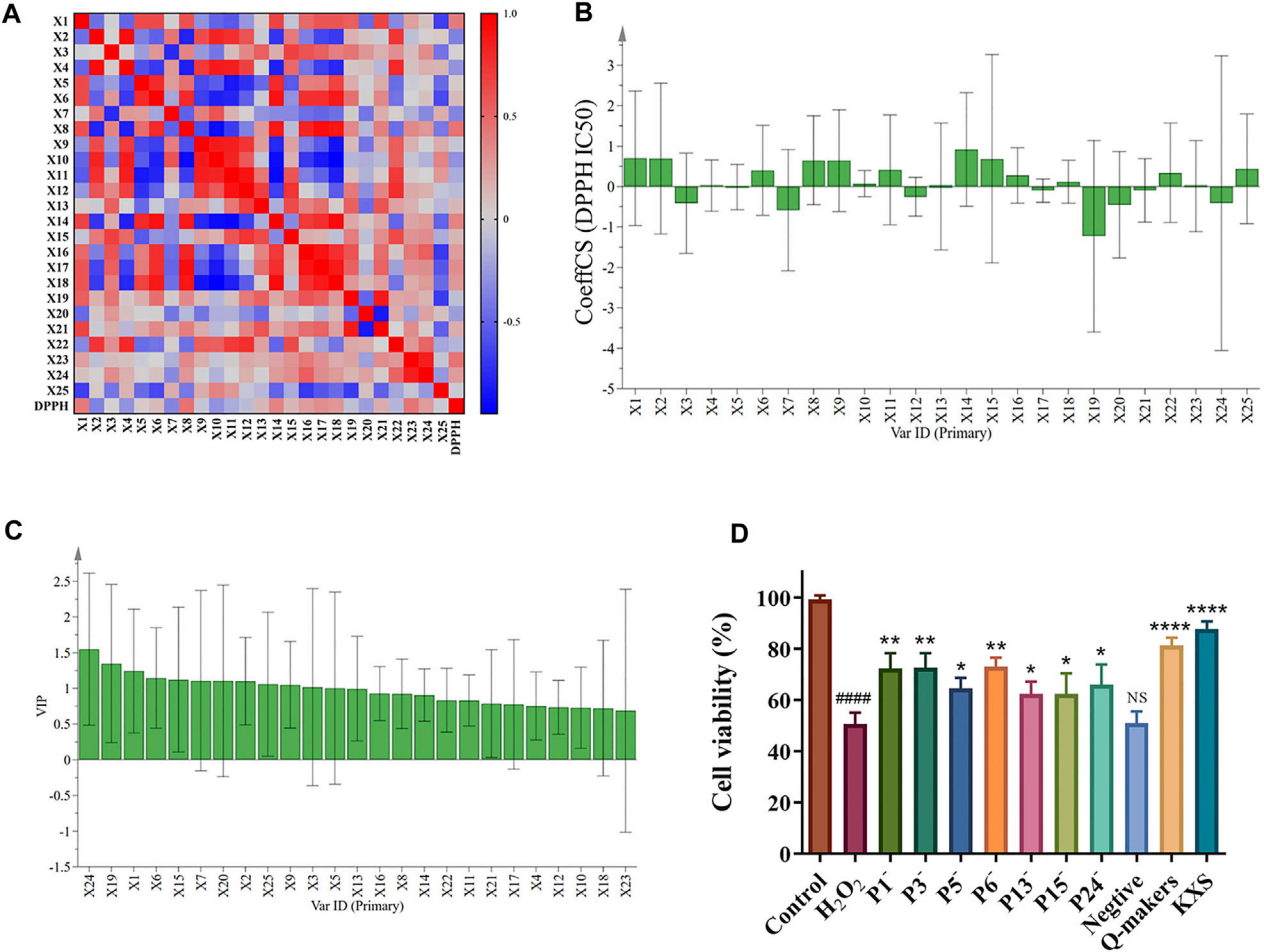
Results of Pearson correlation analysis **(A)**, standardized regression coefficient of scavenging activity ratio of 25 common peaks **(B)**, the VIP contribution of 25 common peaks to the antioxidant activity of KXS extracts **(C)**, and the cell viability of H_2_O_2_-induced SH-SY5Y cells after treating with the different knock-outed KXS samples **(D)**. (P1^-^/ P3^-^/ P5^-^/ P6^-^/ P13^-^/ P15^-^/ P24^-^: the samples knocked out peak 1, 3, 5, 6, 13, 15, 24, respectively; negative: the sample knocked out all 1, 3, 5, 6, 13, 15, and 24 peaks; Q-makers: the sample containing peak 1, 3, 5, 6, 13, 15, and 24). Significantly different compared to the control group: ^####^ *p* < 0.0001; compared to the H_2_O_2_ group: ^****^ *p* < 0.0001, ^**^ *p* < 0.01, ^*^ *p* < 0.05; NS: no significant difference.

The results of Pearson correlation analysis are shown in Figure 3A, where red and blue represent positive and negative correlations, respectively. It can be seen that peaks 8, 1, 14, 17, 18, 24, 16, 21, 15, 13, 6, 5, and 3 in KXS were positively correlated with the DPPH free radical scavenging capacity, suggesting that these metabolites were the main bioactive metabolites for the DPPH free radical scavenging capacity of KXS.

### 3.6 Results of GRA

The correlation results of GRA analysis are listed in Table 4. Subsequently, the ranking results of GRA were as follows: X14 > X8 > X1 > X5 (Sibiricose A6) > X24 > X17 > X18 > X6 > X16 > X13 (Ginsenoside Rg1) > X19 (β-asarone) > X23 > X15 > X11 (3’, 6-disinapoylsucrose) > X7 > X12 > X20 > X25 > X22 > X9 >X3 > X4 (Sibiricose A5) > X21 (α-asarone) > X2> X10 (Polygalaxanthone III). The importance of common peaks increases with their rank, indicating that this metabolite has a greater contribution to scavenging capacity (Nijat et al., 2021). Furthermore, all relational grades are above 0.6, revealing that the DPPH free radical scavenging capacity is the result of the joint action of multiple metabolites in the KXS formula.

**TABLE 4:** Correlation results between common peaks of the HPLC fingerprint and DPPH free radical scavenging effect of KXS extracts.

Peak number	Correlation coefficient	Peak number	Correlation coefficient
X1	0.761	X14	0.777
X2	0.678	X15	0.732
X3	0.695	X16	0.750
X4	0.687	X17	0.754
X5	0.755	X18	0.752
X6	0.751	X19	0.739
X7	0.712	X20	0.706
X8	0.774	X21	0.706
X9	0.701	X22	0.738
X10	0.674	X23	0.703
X11	0.714	X24	0.755
X12	0.712	X25	0.705
X13	0.739		

### 3.7 Results of OPLS-DA

The normalized regression coefficients of the DPPH free radical scavenging capacity of 25 common peaks in KXS extracts are plotted in Figure 3B. The regression equation for the KXS scavenging DPPH free radical capacity is as follows:

Y = 1.395 + 0.697*X1 + 0.693*X2 − 0.414*X3 + 0.022*X4 − 0.012*X5 + 0.399*X6 − 0.587*X7 + 0.649*X8 + 0.642*X9 + 0.070*X10 + 0.411*X11 − 0.254*X12 + 0.001*X13 + 0.917*X14 + 0.686*X15 + 0.273*X16 − 0.102*X17 + 0.116*X18 − 1.230*X19 − 0.452*X20 − 0.097*X21 + 0.338*X22 + 0.008*X23 − 0.410*X24 + 0.437*X25.

Variable influence on projection (VIP) explains the contribution of the independent variable to the dependent variable. The larger the VIP value, the greater the contribution of the independent variable to the dependent variable. Additionally, VIP> 1 indicates a significant contribution to the dependent variable (Xia et al., 2021). As shown in Figure 3C, the scavenging rate of DPPH free radicals was used as the efficacy index for VIP; VIP of 24, 19, 1, 6, 15, 7, 20, 2, 25, 9, 3, 5, and 13 peaks had significant contributions to the antioxidant effect. According to the contribution degree, the antioxidant capacities of KXS extracts were ranked as follows: X24 > X19 (β-asarone) > X1 > X6 > X15 > X7 > X20 > X2 > X25 > X9 > X3 > X5 (Sibiricose A6) > X13 (Ginsenoside Rg1) > X16 > X8 > X14 > X22 > X11 (3′,6-disinapoylsucrose) > X21 (α-asarone) > X17 > X4 (Sibiricose A5) > X12 > X10 (Polygalaxanthone III) > X18 > X23. By cross-enriching the peaks screened by Pearson correlation analysis, GRA, and OPLS-DA, peaks 1, 3, 5 (Sibiricose A6), 6, 13 (Ginsenoside Rg1), 15, and 24 were consequently obtained, which can be considered the Q-markers of KXS for the DPPH free radical scavenging capacity.

### 3.8 Results of the antioxidant activity test

To further validate the relationship between the screened Q-markers and antioxidant activity, the CCK-8 assay was applied to measure the viability of SH-SY5Y cells before and after KXS treatment. The results in Figure 3D demonstrated that 200 μmol/L of H_2_O_2_ significantly damaged the SH-SY5Y cells after 24 h treatment (*p*<0.0001). The cell viability was increased after treating with the samples that knocked out peaks 1, 3, 5, 6, 13, 15, and 24, respectively (*p* < 0.01, *p* < 0.05). Interestingly, KXS and its Q-markers remarkably reduced the oxidative damage caused by H_2_O_2_ and increased the damaged cell survival (*p* < 0.0001). However, the antioxidant activity of the samples was reduced after these Q-markers were knocked out, respectively. Notably, when all Q-markers were knocked out, there was no significant difference in cell viability in the negative group compared to the H_2_O_2_ group. These results indicated that the Q-markers of KXS screened in this study were the main active substances for the antioxidant activity of KXS.

## 4 Discussion

Recently, the quality evaluation standard of TCM has become the most concerned issue with the widespread application of TCM and the improvement of people’s awareness of drug safety (Ren et al., 2020). To address the common quality control problems in TCM, Academician Liu (Liu et al., 2018b) proposed the concept of Q-marker, which brings a new juncture for the research of quality standards of TCM. However, Q-marker screening for the antioxidant effect of KXS has not been conducted so far. Therefore, the novelties of this study include screening the Q-markers of KXS for antioxidant activity by the spectrum–effect relationship based on the identification of the metabolites in KXS by UPLC-Q-Exactive Orbitrap MS/MS and validating the efficacy of Q-markers at the cellular level. First, we applied UPLC-Q-Exactive Orbitrap MS/MS and identified 103 metabolites in KXS. Subsequently, we established the HPLC fingerprints of 12 batches of KXS from different origins and evaluated their similarity. The results showed that the similarity values between each sample were greater than 0.900, which meant that the quality of KXS was relatively consistent and stable.

It is well known that the ability of scavenging DPPH free radicals is positively correlated with the antioxidant activity of drugs (Chen and Huang, 2019). The quality of Chinese botanical drugs is affected by different factors such as the growing environment, geographical location, cultivation technology, and processing (Liu et al., 2018c; Zhao et al., 2019). In this study, even though all of the KXS contained α-asarone, its content varied from batch to batch. As a result, the SC_50_ values of 12 batches of KXS were in the range of 3.69–37.32 μg/mL, indicating that KXS had a strong free radical scavenging ability with concentration dependence, and the antioxidant activity of KXS was correlated with the content of metabolites (Lee et al., 2016; Li et al., 2022). Meanwhile, these results objectively reflect the necessity of establishing a stable quality control system for scientific development of TCM. This is exactly the reason why we screen the Q-markers of KXS to more comprehensively control its quality and ensure its efficacy in the future.

The Pearson correlation analysis and GRA are multi-factor statistical analysis that reflects the correlation degree of elements between two systems (Murali et al., 2022). The results in this study showed that the correlations between the peak areas of the common peaks in KXS and their DPPH free radical scavenging activity were all greater than 0.6, indicating that multiple metabolites in KXS were associated with free radical scavenging activity. In particular, the peaks of 8, 1, 14, 17, 18, 24, 16, 21, 15, 13, 6, 5, and 3 from KXS were positively correlated with the DPPH scavenging activity. However, the Pearson correlation analysis and GRA mainly reflect the association between elements in the system and lack the overall description. Fortunately, the OPLS-DA method can compensate for this deficiency (Genisheva et al., 2018). In the current study, OPLS-DA was also applied to analyze the DPPH radical scavenging activity of 12 batches of KXS. The OPLS-DA results revealed that VIP of peaks 24, 19, 1, 6, 15, 7, 20, 2, 25, 9, 3, 5, and 13 was higher than 1, suggesting that these metabolites had significant contributions to its antioxidant effect (Gao et al., 2019; Wang et al., 2020). Combining with the aforementioned results of three analyses and taking the common intersection of the screened peaks, the Q-markers of KXS for the DPPH free radical scavenging activity were obtained as peaks 1, 3, 5 (Sibiricose A6), 6, 13 (Ginsenoside Rg1), 15, and 24. To further validate the Q-markers of KXS for antioxidant activity, the protective effect of the Q-marker sample and the Q-marker-knockout samples on H_2_O_2_-induced SH-SY5Y cells was detected by the CCK-8 method (Choi et al., 2023). Our results showed that KXS and its Q-markers remarkably reduced the SH-SY5Y cell damage caused by H_2_O_2_. However, the antioxidant activity of Q-markers of completely or partially knocked out samples was significantly reduced compared to KXS. These results indicated that the Q-markers of KXS screened in this study were the main bioactive metabolites for the antioxidant activity of KXS.

Because of the extensive sources and complex metabolites, it is difficult to determine the pharmacodynamic material basis of TCM (Liang et al., 2022). Comfortingly, the spectrum–effect relationship has become an important tool for exploring the relationship between the pharmacological effects and the material basis of TCM (Rao et al., 2022). It organically connects the fingerprint (active metabolites) and pharmacological data on TCM, and systematically reveals the relationship between them through a reasonable spectrum–effect model and analytical method (Wang et al., 2019c). In our research, the Pearson correlation analysis, GRA, and OPLS-DA were innovatively combined to investigate the spectrum–effect relationship between the HPLC fingerprint and the antioxidant activity of KXS extracts. The results revealed that the antioxidant activity of KXS was a comprehensive representation of various metabolites. Our analysis suggests that peaks 1, 3, 5, 6, 13, 15, and 24 are the potential antioxidant Q-markers of KXS, among which peaks 5 and 13 are Sibiricose A6 and Ginsenoside Rg1, respectively. Certainly, the metabolites of KXS are complex, and other metabolites needed to be further identified in the future.

## 5 Conclusion

In summary, 103 metabolites were identified from KXS by UPLC-MS/MS. The typical HPLC fingerprints of 12 batches of KXS extracts were established in this study, and the similarity values were in the range of 0.911–0.999. Meanwhile, the radical scavenging activity of 12 batches of KXS was determined by the DPPH method. Their SC_50_ values ranged from 3.69 to 37.32 μg/mL, with a definite concentration–effect relationship. Combined with the Pearson correlation analysis, GRA, and OPLS-DA, the potential antioxidant Q-markers in KXS were screened, which are peaks 1, 3, 5 (Sibiricose A6), 6, 13 (Ginsenoside Rg1), 15, and 24 in its HPLC fingerprints. Finally, the antioxidant activity of Q-markers of KXS was validated in H_2_O_2_-induced SH-SY5Y cells. Here, the identification of KXS metabolites and its spectrum–antioxidant effect relationship provide a pharmacodynamic material basis for its antioxidant efficacy, as well as a scientific reference for its Q-markers and the quality control standards.

## Data Availability

The datasets presented in this study can be found in online repositories. The names of the repository/repositories and accession number(s) can be found in MetaboLights Database MTBLS8806.

## Abbreviations

AUCM Anhui Education Department, AD Alzheimer’s disease, APA Average peak area, DPPH 2,2-Diphenyl-1-picrylhydrazyl, GRA Gray relational analysis, HPLC High-performance liquid chromatography, KXS Kai-Xin-San, OPLS-DA Orthogonal partial least squares discriminant analysis, Q-marker Quality marker, RSD Relative standard deviation, RT Retention time, TCM Traditional Chinese medicine, VIP Variable influence on projection.
