# Supplementary material for: Research on the quality markers of antioxidant activity of Kai-Xin-San based on the spectrum–effect relationship
Source: Front Pharmacol. 2023 Dec 27;14:1270836. doi: 10.3389/fphar.2023.1270836 (PMC10777484; doi:10.3389/fphar.2023.1270836)
Supplement: Supplementary file 1 [file Table1.DOCX]

**Research on the** **quality markers of antioxidant activity of Kai-Xin-San based on** **spectrum-effect relationship**

Xiaoxiao Shan ^a,b,c,d,e#^, Xuan Yang ^a,b,c,d,e#^, Dawei Li ^a,b,c,d,e^, Lele Zhou ^a,b,c,d,e^, Shaogang Qin ^f^, Junying Li ^a,b,c,d,e^, Wenkang Tao ^a,b,c,d,e^, Can Peng ^a,b,c,d,e^, Jinming Wei ^a,b,c,d,e^, Xiaoqin Chu ^a,b,c,d,e*^, Haixuan Wang ^f*^, Caiyun Zhang ^a,b,c,d,e*^

(a Anhui Province Key Laboratory of Pharmaceutical Preparation Technology and Application, Anhui University of Chinese Medicine, Hefei 230012, Anhui, China; b Engineering Technology Research Center of Modernized Pharmaceutics, Anhui Education Department (AUCM), Hefei 230012, Anhui, China; c School of Pharmacy, Institute of Pharmacokinetics, Anhui University of Chinese Medicine, Hefei 230012, Anhui, China; d Anhui Genuine Chinese Medicinal Materials Quality Improvement Collaborative Innovation Center, Hefei 230012, Anhui, China; e Anhui Academy of Chinese Medicine, Anhui University of Chinese Medicine, Hefei 230012, P.R. China; ^f^ Hefei Food and Drug Inspection Center Hefei 230001, Anhui, China)

# Xiaoxiao Shan and Xuan Yang contributed to equally to this paper and should be regarded as co-first authors.

*Corresponding authors.
*Address correspondence to [chuxq420@163.com](mailto:chuxq420@163.com); 114373238@qq.com; cyzhang6@ustc.edu.cn.

Table S1 Raw material source information for 12 batches of KXS

| Serial Number | *Polygala tenuifolia Willd.* | | *Panax ginseng C. A. Mey.* | | *Poria cocos* | | *Acorus tatarinowii Schott* | |
| --- | --- | --- | --- | --- | --- | --- | --- | --- |
|  | Origin | Batch number | Origin | Batch number | Origin | Batch number | Origin | Batch number |
| S1 | Ruicheng Shanxi | YZ202201 | Baishan Jilin | RS202201 | Tengchong Yunnan | FL202101 | Jinzhai Anhui | SCP202201 |
| S2 | Guxian Shanxi | YZ202202 | Yanji Jilin | RS202202 | Jizhai Anhui | FL202102 | Shucheng Anhui | SCP202202 |
| S3 | Xinjiang Shanxi | YZ202203 | Baishan Jilin | RS202201 | Tengchong Yunnan | FL202101 | Jinzhai Anhui | SCP202201 |
| S4 | Ruicheng Shanxi | YZ202201 | Yanji Jilin | RS202202 | Tengchong Yunnan | FL202101 | Jinzhai Anhui | SCP202201 |
| S5 | Ruicheng Shanxi | YZ202201 | Changbaishan Jilin | RS202203 | Tengchong Yunnan | FL202101 | Jinzhai Anhui | SCP202201 |
| S6 | Ruicheng Shanxi | YZ202201 | Baishan Jilin | RS202201 | Tengchong Yunnan | FL202101 | Shucheng Anhui | SCP202202 |
| S7 | Ruicheng Shanxi | YZ202201 | Baishan Jilin | RS202201 | Huoshan Anhui | FL202104 | Jinzhai Anhui | SCP202201 |
| S8 | Ruicheng Shanxi | YZ202201 | Yanji Jilin | RS202202 | Jinzhai Anhui | FL202102 | Shucheng Anhui | SCP202202 |
| S9 | Xinjiang Shanxi | YZ202203 | Yanji Jilin | RS202202 | Jinzhai Anhui | FL202102 | Shucheng Anhui | SCP202202 |
| S10 | Guxian Shanxi | YZ202202 | Yanji Jilin | RS202202 | Jinzhai Anhui | FL202102 | Jinzhai Anhui | SCP202201 |
| S11 | Ruicheng Shanxi | YZ202201 | Changbaishan Jilin | RS202203 | Huoshan Anhui | FL202104 | Ji’an Jiangxi | SCP202203 |
| S12 | Xinjiang Shanxi | YZ202203 | Changbaishan Jilin | RS202203 | Huoshan Anhui | FL202104 | Jinzhai Anhui | SCP202201 |

Table S2 The metabolites identified in the KXS

| **(A) positive ion mode** | | | | | |
| --- | --- | --- | --- | --- | --- |
| **No.** | **Components** | **Molecular formula** | **ESI^+^, m/z** | **RT (min)** | **Origin** |
| 1 | Spermidine | C_7_H_19_N_3_ | 146.16495[M+H]^+^ | 0.94 | B |
| 2 | Spermine | C_10_H_26_N_4_ | 203.22244[M+H]^+^, 129.13846, 112.11193 | 1.007 | B |
| 3 | fructose | C_6_H_12_O_6_ | 215.03329[M+CI]^-^, 113.02489, 101.02484, 95.0140, 89.02474 | 1.104 | B |
| 4 | Adenine | C_5_H_5_N_5_ | 136.06140[M+H]^+^, 119.03549 | 1.108 | BD |
| 5 | Aristolactam AII | C_16_H_11_NO_3_ | 266.12302[M+H]^+^, 248.11258, 230.10216 | 1.369 | A |
| 6 | Tenuifoliose H | C_61_H_74_O_34_ | 1351.41150[M+H]^+^ | 1.39 | A |
| 7 | Pantothenic acid | C_9_H_17_NO_5_ | 220.11754[M+H]^+^ | 1.425 | B |
| 8 | Anethole | C_10_H_12_O | 149.09595[M+H]^+^ | 1.614 | B |
| 9 | Uridine | C_9_H_12_N_2_O_6_ | 243.06290[M-H]^-^, 243.06352, 111.02044, 82.03015, 68.01442 | 1.776 | BD |
| 10 | Geraldone | C_16_H_12_O_5_ | 143.03891[M+2H]^2+^, 143.03372, 120.02348 | 3.777 | D |
| 11 | Asaronaldehyde | C_10_H_12_O_4_ | 197.08066[M+H]^+^, 182.05739,169.08591, 139.03847 | 8.348 | C |
| 12 | Blumenol A | C_13_H_20_O_3_ | 225.14838[M+H]^+^, 193.08578 | 8.591 | C |
| 13 | Anisaldehyde | C_8_H_8_O_2_ | 137.05946[M+H]^+^ | 9.357 | B |
| 14 | α-Ionone | C_13_H_20_O | 193.15843[M+H]^+^, 135.11691 | 9.58 | C |
| 15 | Tenuifoliose I | C_59_H_72_O_33_ | 1309.40161[M+H]^+^ | 10.355 | A |
| 16 | 1-Hydroxyacoronene | C_15_H_22_O_3_ | 251.16373[M+H]^+^, 125.05956, 233.15363 | 10.414 | C |
| 17 | (E)-3,4,5-Trimethoxycinnamic acid | C_12_H_14_O_5_ | 239.09116[M+H]^+^, 221.08052, 193.08553, 191.03568, 190.06337 | 10.645 | A |
| 18 | 1-Hydroxy-3,7-dimethoxyxanthone | C_15_H_12_O_5_ | 273.07547[M+H]^+^, 153.01822 | 11.807 | A |
| 19 | 6-Hydroxy-1,2,3,7-tetramethoxy-9H-xanthen-9-one | C_17_H_16_O_7_ | 333.09656[M+H]^+^, 318.07303 | 12.088 | A |
| 20 | Tenuifoliose A | C_62_H_76_O_35_ | 1381.42273[M+H]^+^ | 12.465 | A |
| 21 | Kaempferol | C_15_H_10_O_6_ | 287.05466[M+H]^+^, 272.03128 | 12.802 | C |
| 22 | Paeonol | C_9_H_10_O_3_ | 167.07004[M+H]^+^, 149.06023 | 13.913 | BCD |
| 23 | Grossamide | C_36_H_36_N_2_O_8_ | 625.25317[M+H]^+^, 488.16699, 121.06472 | 14.748 | C |
| 24 | 1,2,3,6,7-Pentamethoxyxanthone | C_18_H_18_O_7_ | 347.11221[M+H]^+^, 332.0889 | 15.173 | AB |
| 25 | 1,3-Dihydroxy-4,5-dimethoxyxanthone | C_15_H_12_O_6_ | 289.07028[M+H]^+^, 274.04715 | 15.323 | A |
| 26 | Euphorbia factor I | C_36_H_58_O_6_ | 587.42975[M+H]^+^, 177.05455 | 17.489 | BD |
| 27 | Ginsenoside Rh3 | C_36_H_60_O_7_ | 605.44006[M+H]^+^, 425.37863, 191.17986, 109.10091 | 17.499 | B |
| 28 | 1,7-Dimethoxyxanthone | C_15_H_12_O_4_ | 257.08063[M+H]^+^, 242.05717 | 17.623 | AD |
| 29 | Onjisaponin G | C_70_H_104_O_32_ | 1457.65515[M+H]^+^ | 18.208 | A |
| 30 | 4-tert-Octylphenol | C_14_H_22_O | 189.16362[M+H-H2O]^+^, 133.10114 | 18.827 | C |
| 31 | Dihydro-resveratrol | C_14_H_14_O_3_ | 231.10144[M+H]^+^, 121.13583, 138.06703 | 20.115 | B |
| 32 | 1,6-Dihydroxy-3,5,7-trimethoxyxanthone | C_16_H_14_O_7_ | 319.08090[M+H]^+^, 304.05661 | 20.18 | A |
| 33 | 2,4-Dimethylcinnamic acid | C_11_H_12_O_2_ | 209.11661[M+H+MeOH]^+^, 177.09096, 121.06461 | 21.109 | C |
| 34 | Onjisaponin E | C_71_H_106_O_33_ | 1487.66931[M+H]^+^ | 21.43 | A |
| 35 | Altechromone A | C_11_H_10_O_3_ | 191.07018[M+H]^+^ | 21.628 | B |
| 36 | N, N-Diethylbenzylamine | C_11_H_17_N | 164.14304[M+H]^+^ | 22.864 | C |
| 37 | β-Asarone | C_12_H_16_O_3_ | 209.11699[M+H]^+^, 194.09360, 181.08580, 179.07077 | 24.163 | C |
| 38 | α-Asarone | C_12_H_16_O_3_ | 209.11794[M+H]^+^, 194.09418, 181.08717, 179.07202 | 24.339 | C |
| 39 | α-Humulene epoxide | C_15_H_24_O | 221.18983[M+H]^+^, 203.17937 | 24.501 | BC |
| 40 | Gomisin N | C_23_H_28_O_6_ | 401.19522[M+H]^+^, 181.08577 | 26.018 | B |
| 41 | (9r,10s)-epoxyheptadecan-4,6-diyn-3-one | C_17_H_24_O_2_ | 261.18518[M+H]^+^, 105.03345 | 26.953 | B |
| 42 | Poricoic acid D | C_31_H_46_O_6_ | 515.33624[M+H]^+^, 497.32925, 479.31924 | 27.33 | D |
| 43 | 1-Hydroxy-3,6,7-trimethoxy xanthone | C_16_H_14_O_6_ | 303.08597[M+H]^+^, 288.06314 | 28.099 | A |
| 44 | Ginsenoyne D | C_17_H_26_O_2_ | 295.22650[M+H+MeOH]^+^, 295.22678, 277.21628 | 28.829 | B |
| 45 | Acoramol | C_12_H_16_O_4_ | 225.11208[M+H]^+^, 210.08875, 193.08592, 165.09102 | 33.178 | C |
| 46 | (3Z)-Hex-3-en-1-ol | C_6_H_12_O | 101.09598[M+H]^+^ | 33.294 | A |
| 47 | N,N-Dimethyldecanamide | C_12_H_25_NO | 200.20082[M+H]^+^ | 35.095 | B |
| 48 | 1(10)-Aristolen-2-one | C_15_H_22_O | 219.17421[M+H]^+^, 149.09619 | 36.21 | C |
| 49 | (E)-4-Methoxycinnamic acid | C_10_H_10_O_3_ | 179.07022[M+H]^+^, 147.04411 | 36.948 | C |
| 50 | Poricoic acid B | C_30_H_44_O_5_ | 485.32602[M+H]^+^, 467.35233, 439.35599 | 37.643 | D |
| 51 | Dehydrotumulosic acid | C_31_H_46_O_6_ | 485.36206[M+H]^+^, 467.35156, 449.34076, 311.23694, 293.22620 | 37.716 | D |
| 52 | 3,5-Dimethyl-4-methoxybenzoic acid | C_10_H_12_O_3_ | 181.08582[M+H]^+^, 166.06253, 151.07555 | 37.977 | B |
| 53 | Tumulosic acid | C_31_H_50_O_4_ | 487.37769[M+H]^+^, 469.36804 | 38.272 | D |
| 54 | Dehydroeburicoic acid | C_31_H_48_O_3_ | 469.36691[M+H]^+^, 451.35648 | 38.274 | D |
| 55 | Elemicin | C_12_H_16_O_3_ | 209.11713[M+H]^+^, 194.09378, 168.07819 | 38.521 | C |
| 56 | Veraguensin | C_22_H_28_O_5_ | 373.20081[M+H]^+^,235.13324,179.10661,151.07516 | 38.586 | C |
| 57 | 3,4-Dihydrocadalene | C_15_H_20_ | 201.16379[M+H]^+^,159.11685,145.10121 | 38.727 | C |
| 58 | (Z)-methylisoeugenol | C_11_H_14_O_2_ | 179.10657[M+H]^+^, 151.07536 | 38.826 | C |
| 59 | Poricoic acid G | C_30_H_46_O_5_ | 487.34183[M+H]^+^, 469.36282, 313.25342 | 39.27 | D |
| 60 | Polyporenic acid C | C_31_H_46_O_4_ | 483.34659[M+H]^+^, 465.33542 | 39.648 | D |
| 61 | Dibutyl phthalate | C_16_H_22_O_4_ | 279.15906[M+H]^+^, 279.09488, 150.02620, 149.02333 | 40.015 | B |
| 62 | Naphthalene,1,2,3,4-tetrahydro-1,5,7-trimethyl | C_13_H_18_ | 175.14798[M+H]^+^, 133.1012 | 40.466 | C |
| 63 | Falcarinol | C_17_H_24_O | 262.21631[M+NH_4_]^+^, 143.08542 | 40.511 | B |
| 64 | Oleic acid | C_18_H_34_O_2_ | 283.26297[M+H]^+^, 97.10113 | 41.415 | D |
| 65 | Methyl geranate | C_11_H_18_O_2_ | 183.13776[M+H]^+^, 113.05961, 71.01277 | 41.44 | D |
| 66 | δ-Elemene | C_15_H_24_ | 205.19495[M+H]^+^, 191.14359, 163.14824, 149.13252, 123.11694, 97.10113, 83.08543 | 41.472 | B |
| 67 | δ-7-Stigmasterol | C_29_H_46_O | 411.36041[M+H]^+^ | 43.37 | D |
| 68 | Ginsenoyne C | C_17_H_24_O_3_ | 309.20593[M+H+MeOH]^+^ | 43.778 | B |
| 69 | Pachyman | C_33_H_52_0_5_ | 529.38818[M+H]^+^, 163.14795 | 45.605 | D |
| 70 | α-Spinasterol | C_29_H_48_O | 413.37711[M+H]^+^, 395.36630, 255.21169 | 46.23 | AB |
| 71 | Cymol | C_10_H_14_ | 135.11687[M+H]^+^ | 46.26 | C |
| 72 | 8-(3-Heptyloxiran-2-yl)-1-methoxyocta-4,6-diyn-3-one | C_18_H_26_O_3_ | 291.19531[M+H]^+^, 161.0602 | 46.358 | B |
| 73 | 13-Tetradecenyl acetate | C_16_H_30_O_2_ | 255.23166[M+H]^+^, 221.03345,203.02251 | 46.517 | B |
| 74 | cis-9,cis-12-linoleic acid | C_18_H_32_O_2_ | 281.24722[M+H]^+^, 263.23376, 245.22597 | 48.294 | BD |
| 75 | o-acetylpachymicacid-25-ol | C_35_H_56_O_7_ | 571.39807[M+H-H2O]^+^ | 48.502 | D |
| **(B) Negative ion mode** | | | | | |
| **No.** | **Components** | **Molecular formula** | **ESI^+^, m/z** | **RT (min)** | **Origin** |
| 1 | D-(-)-Mannitol | C_6_H_14_O_6_ | 181.07237[M-H]^-^, 163.06194, 119.03525, 89.02476 | 1.024 | C |
| 2 | D-Mannuronic acid | C_6_H_10_O_7_ | 193.03590[M-H]^-^, 103.00404 | 1.028 | B |
| 3 | Sucrose | C_12_H_22_O_11_ | 387.11496[M+FA-H]^-^, 341.11026, 179.05646, 119.03525, 89.02464, 71.01404, 59.01400 | 1.042 | ABD |
| 4 | D-(−)-Quinic acid | C7H12O6 | 191.05644[M-H]^-^, 173.04601, 155.03549, 127.04051, 111.04554, 85.02976 | 1.1 | D |
| 5 | polygalitol | C_6_H_12_O_5_ | 209.06691[M+FA-H]^-^, 101.02473, 87.00920, 57.03479 | 1.113 | AB |
| 6 | coriose | C_7_H_14_O_7_ | 209.06708[M-H]^-^, 85.02988 | 1.244 | D |
| 7 | (±)-Malic Acid | C_4_H_6_O_5_ | 133.01453[M-H]^-^, 133.01459, 115.00400, 71.01406 | 1.281 | B |
| 8 | Pantothenic acid | C_9_H_17_NO_5_ | 218.10429[M-H]^-^, 146.08299, 88.04081 | 3.503 | B |
| 9 | Tenuifoliose N | C_63_H_78_O_36_ | 1409.42212[M-H]^-^ | 1.492 | A |
| 10 | Malonic acid | C_3_H_4_O_4_ | 103.00408[M-H]^-^, 103.00415, 102.93209, 59.01409, 57.03477 | 1.525 | B |
| 11 | Succinic acid | C_4_H_6_O_4_ | 117.01974[M-H]^-^, 117.01977, 99.00916, 73.02978, 55.01913 | 1.977 | B |
| 12 | Dimethyl L-malate | C_6_H_10_O_5_ | 161.04617[M-H]^-^, 143.03572, 89.02488 | 2.134 | CD |
| 13 | Gallic Acid | C_7_H_6_O_5_ | 169.01489[M-H]^-^, 125.02493 | 2.311 | D |
| 14 | Glucogallin | C_13_H_16_O_10_ | 331.06848[M-H]^-^, 169.01476, 125.02509 | 2.663 | D |
| 15 | Taxifolin | C_15_H_12_O_7_ | 303.05103[M-H]^-^ | 3.858 | B |
| 16 | sibiricose A5 | C_22_H_30_O_14_ | 517.15680[M-H]^-^, 337.09589, 193.05042, 175.04010 | 6.055 | A |
| 17 | Sibiricose A6 | C_23_H_32_O_15_ | 547.16736[M-H]^-^ | 7.624 | A |
| 18 | Tenuifoliose P | C_59_H_74_O_34_ | 1325.39502[M-H]^-^ | 8.2 | A |
| 19 | Polygalaxanthone III | C_25_H_28_O_15_ | 567.13550[M-H]^-^, 435.09213, 345.06171, 315.05081, 272.03253 | 13.061 | A |
| 20 | Benzoic acid | C_7_H_6_O_2_ | 121.03002[M-H]^-^ | 13.472 | ACD |
| 21 | 3^’^,6-Disinapoyl sucrose | C_34_H_42_O_19_ | 753.20166[M-H]^-^, 753.22168, 547.16693, 223.05946 | 20.100 | A |
| 22 | 1,6-Dihydroxy-3,5,7-trimethoxyxanthone | C_16_H_14_O_7_ | 317.06796[M-H]^-^, 287.02097, 259.02588 | 20.028 | A |
| 23 | Methyl dehydroabietate | C_21_H_30_O_2_ | 313.21631[M-H]^-^, 183.14063 | 29.619 | D |
| 24 | Ginsenoside Rg1 | C_42_H_72_O_14_ | 845.49091[M+FA-H]^-^, 799.48535, 637.43530, 475.37888 | 30.01 | B |
| 25 | 3-Epidehydrotumulosic acid | C_31_H_48_O_4_ | 483.35040[M-H]^-^, 483.35016, 439.35876, 337.25565 | 40.542 | D |
| 26 | Pachymic acid | C_33_H_52_O_5_ | 527.37280[M-H]^-^, 465.34296 | 44.221 | D |
| 27 | 20(R)-Ginsenoside-Rh2 | C_36_H_62_O_8_ | 621.43616[M-H]^-^ | 44.28 | B |
| 28 | Ginsenoside-Rk3 | C_36_H_60_O_8_ | 619.42157[M-H]^-^ | 44.652 | B |

A: Polygalae Radix B: Ginseng Radix et Rhizoma C: Acori Tatarinowii Rhizoma D: Poria cocos
